# Living environment and health of under-five children in urban slums of a coastal region in South India

**DOI:** 10.4314/gmj.v54i4.6

**Published:** 2020-12

**Authors:** VR Roja, Prakash Narayanan, Varalakshmi Chandra Sekaran, MG Ajith Kumar

**Affiliations:** 1 ICMR NTF HI project, Dept. of ENT & HNS, AIIMS Raipur, India; 2 Prasanna School of Public Health, MAHE, Manipal, India; 3 Department of Community Medicine, Melaka Manipal Medical College, MAHE, Manipal, India; 4 Baby Memorial Hospital and Malabar Hospital, Calicut, Kerala, India

**Keywords:** Child morbidity, under-five children, hygiene, slums, India

## Abstract

**Objective:**

The primary objective of the study was to determine the association between the living environment and morbidity, nutritional status, immunization status, and personal hygiene of under-five children living in urban slums in southern India.

**Methods:**

This study included 224 mothers of under-five children living in urban slums of Udupi Taluk, Karnataka. A total of 17 urban slums were selected randomly using random cluster sampling.

**Results:**

Undernutrition was high among children of illiterate mothers (63.8%), and the children of working mothers were affected by more morbidity (96.6%) as compared with housewives. Morbidity was also found to be high among children belonging to families with low incomes (66.1%) and low socio-economic backgrounds (93.1%). Safe drinking water, water supply, sanitation, hygiene, age of the child, mother's and father's education, mother's occupation and age, number of children in the family, use of mosquito nets, type of household, and family income were significantly associated with child morbidity, nutritional status, immunization status, and personal hygiene of under-five children living in urban slums.

**Conclusion:**

Overall, in our study, family characteristics including parental education, occupation and income were significantly associated with outcomes among under-five children. The availability of safe drinking water and sanitation, and the use of mosquito nets to prevent vector-borne diseases are basic needs that need to be urgently met to improve child health.

**Funding:**

Self-funded

## Introduction

According to the constitution of the World Health Organization 1948 “Healthy development of the child is of basic importance; the ability to live harmoniously in a changing total environment is essential to such development”.^1^ As cities develop, urbanization is emerging as a public health challenge. There has been consistent growth in the urban population from 18% to 33% between 1955 and 2015. Urbanization leads to the establishment of slums, which continue to lack basic sanitary facilities and safe drinking water, healthcare, housing needs, and educational deficits which pose as growing concerns. The lack of these basic services has both a direct and indirect effect on the health of the urban slum dwellers2. The Sustainable Development Goals^2^ through^6^ West Bengal, Rajasthan and Gujarat which enumerated more than 81% slum population and 1955 slum towns^4^. There is a steady growth in the slum population in the developing world with an estimated 881 million in 2014 in comparison with 792 million in the year 2000. Per the Census of India, 2011^5^,^6^ several Indian states including Karnataka contribute to about 1955 slum towns. It is also estimated that every fourth Indian child lives in an urban setting. The number of children between the ages of 0–6 years in urban areas has shown an increase since 2001 while a steady decline has been simultaneously observed in rural areas^7^. About 7.8 million children, accounting for 6.07%, live in urban slums.^8^ Children living in slums are 1.3 times likely to suffer from diarrhoea than those in non-slum areas.^9^ About 300 million people living in urban India face exclusion from essential health services and other services like safe drinking water, sanitation, education, and access to essential services and most often their births or deaths are not registered. Children are prone to diseases and disasters.^2^,^9^–^14^. According to the National Family and Health Survey (NFHS)-4 only 63.9% of the urban poor children get fully immunized, 49.8% of urban poor under-five children were underweight.^2^,^10^ The sustainable development goals (SDG) envisage the end of malnutrition among the most vulnerable by the year 2030 and the World Health Organization hopes to reduce childhood wasting below 5%.^12^

On assessing the Global Hunger Index reflecting severity in hunger levels, undernourishment, child wasting, stunting, and mortality, India's performance begs further efforts^15^. Slum-dwellers are largely migrants and are mobile who live in unhygienic and insanitary conditions. Coupled with lower educational status, reduced awareness on health and nutrition and comparatively reduced access to healthcare, this poses a risk of increased morbidity among the most vulnerable groups, including women and children. There is a paucity of literature related to the health of urban slum dwellers, especially of children. In this regard, our study aimed to estimate the morbidity, nutritional and immunization status and personal hygiene of children living in urban slums and their association if any with their living conditions, socio-economic and demographic characteristics of the slum population in Udupi taluk.

## Methods

Study design: This community-based cross-sectional study was designed to study the association of the living environment on child morbidity, nutrition, immunization status, and personal hygiene of under-five children living in urban slums of Udupi taluk, Karnataka.

### Sampling

Random sampling was used to select slums. Seven slums were randomly selected from a list of 27 slums collated from the Municipality and Mission Indradhanush16. This was a mission launched by the Ministry of Health and Family Welfare (MOHFW) Government of India. Cluster sampling technique was used for the selection of households and children in selected slums. A complete enumeration of eligible mothers of under-five children from all selected clusters. If there were more than one eligible under-five children in the selected household, data on the youngest child was collected. The sample size was calculated using the formula for cross-sectional studies^17^, using Z*α* = 1.96, p= 43% was the prevalence from previous study^18^, q was 1-p, d was 15% relative precision as the researcher wanted to estimate the prevalence to within 5 percentage points of the true value at 95% confidence and therefore, the sample size was 224.

### Study participants

A total of 17 urban slums were selected randomly and complete enumerations of all mothers of under-five children from all selected slums were carried out. Mothers of children who were absent during more than two consecutive visits were excluded from the study.

### Ethical considerations

Ethical clearance was obtained from the Institutional Ethical Committee at a tertiary care hospital in Manipal (IEC 438/2015). Written consent was obtained from all the participants in the study.

### Data collection

A structured questionnaire was prepared following review of the literature and this was used for the collection of data. The tool was self-administrated and the interviewer was available to clarify any questions that the interviewee may have had. Nutritional status of under-five children was assessed by including anthropometric measurements like height, weight, and Z-scores were calculated according to National Centre for Health Statistics (NCHS) reference data for age and sex of the child endorsed by the World Health Organiztion.^18^

Children were classified as underweight and normal weight. Undernutrition was the weight for age less than - 2SD of the NFHS reference^18^. The weight of the underfive children was measured using a digital weighing machine. To ascertain the information about immunization coverage, the respondent was asked to provide their immunization card if they had any. In the case of non-availability of the card, information regarding the administration of vaccines was recorded on the basis of the respondent's memory. For BCG, the immunization status was assessed by the presence of the scar.

For this study, morbidity conditions were assessed using the presence of one or more conditions such as skin infections, diarrhoea, fever, cough, pneumonia, eye or ear infections, angular stomatitis, and caries. Personal hygiene was measured using general appearance, bathing practices, hair, nose, mouth, tongue if white coated, teeth and nail hygiene. The living environment of a child was defined as all external factors which may affect the normal growth of child including housing, safe drinking water, sanitation and socio-demographic profile of the parents.^11^

### Data analysis

The data was entered and analyzed in SPSS version 15. The data were summarized using descriptive statistics. Statistical differences between categorical variables were assessed using the Chi-square or Fisher exact test (if cell value was less than 5) and means were compared using the Student's T-test. The p-value <0.05 was considered statistically significant.

## Results

A total of 224 mothers of under-five children participated in this study, the mean age of children was 28±1.6 months and the majority were boys (58.5%). The mean age of father was 32.4±2.5 years and mothers' mean age was 22.9±4.3 years.

Most of the parents were daily labourers. More than two-thirds (67.4%) of the families had only one under-five child, and 85.7% were Hindus. About 38% had a monthly family income below Rs.5000 ($68), and 88% of children were living in kutcha (mud bricks) households. Most (84.4%) of the slums had drinking water supply and more than three-fourths (76%) of the households did not purify or boil their drinking water. None of the households had a toilet facility within the premises, and 64.7% practised open defecation.

On assessing under-five morbidity by age group (in months), it was observed that the highest percentage of morbidities (31%) were among children aged 23–35 months, followed by 29% among children aged 12–22 months with the least reported among those less than 12 months of age (11%).

Most common morbidities identified were skin infections and cough which constituted 45.1% and 44.6% respectively followed by fever (30.8%), pneumonia (27.7%), diarrhoea (24.1%), injuries (22.8%), angular stomatitis (21.9%), ear infection (21.4%) and eye infection (7.6%). Majority of the children (59.8%) aged between 12 to 35 months were suffering from various morbidities. Morbidity was significantly high among young children, whose mothers were younger (p=0.042) and children of illiterate women (p=0.039). Child morbidity was found to be significantly associated working status of mother (p =0.001) and those having a family income of more than Rs 5000 per month and those living in kutcha households (p=0.001).

Morbidity conditions were found to be significantly higher among those who did not use safe or potable drinking water (p=0.009). Significant variations between morbidity and inadequate water supply were noted (p=0.001). Morbidity was high among children who were not using mosquito nets (p=0.021). Morbidity was significantly associated with personal hygiene (p=0.001) as 83% of the under-five morbid children were not having good personal hygiene. The study showed that personal hygiene was found to be significantly associated with skin infections (p=0.041).

The majority (63%) of children with skin infections had poor personal hygiene. Personal hygiene was significantly associated with cough (p=0.003) with almost half (49.7%) of the children who had poor personal hygiene, suffering from a cough. Among the children who did not have adequate personal hygiene, 26.6% had diarrhoea, 30.5% had pneumonia, 35% had fever, 24.3% had angular stomatitis and 22% had ear infections respectively. Children with good personal hygiene showed a smaller number of eye infections (8.1%), dental caries (23.4%) and injuries.

This study shows that undernutrition had a significant association with mothers' education (p=0.039). The proportion of undernourished children was higher among those whose mothers were illiterate (76.7%) as compared to those who were literate. Undernutrition was more among children belonging to families with a family income of less than Rs. 5000 ($68) (78.1%). Undernutrition was significantly associated with family income (p=0.02). Children who were living in kutcha households also had higher rates of undernutrition (97.3%). A significant association between undernutrition and type of household was noted at p=0.001. (Table 1)

**Table 1:** Association between socio-demographic factors and childhood morbidity

Variables	Category	Morbidity		χ^2^	p-value
		Yes	No		
**Child age in** **month**	mean± SD	26.7±1.5	30±1.6	1.56*	**0.214**
**Mother's age**	mean± SD	22.6±4.04	24.08±5.23	4.195*	**0.042**
**Mother's education**	Literate	63(36.20)	10(20.0)	4.644	**0.039**
	Illiterate	111(63.8)	40(80.0)		
**Mother's occupation**	Working	168(96.6)	33(68.0)	39.34	**0.001**
	Housewife	6(3.4)	16(32.0)		
**Monthly income** **(Rs.)**	>5000	115(66.1)	24(48.0)	5.399	**0.020**
	<5000	59(33.9)	26(52.0)		
**Type of** **household**	Semi pucca	10(5.7)	16(32.0)	26.09	**0.001**
	Kutcha	164(94.3)	34(68.0)		
**Purified** **drinking water**	Yes	35 (20.1)	19(38.0)	6.791	**0.009**
	No	139(79.9)	31(62.0)		
**Adequate** **water supply**	Yes	153(87.9)	29(58.0)	22.840	**0.001**
	No	21 (12.1)	21(42.0))		
**Using mosquito** **nets**	Yes	50 (28.7)	10(20.0)	1.511	**0.021**
	No	124(71.3)	40(80.0)		
**Good personal** **hygiene**	Yes	28(16.1)	19(38.0)	11.24	**0.001**
	**No**	**146(83.9)**	**31(62.0)**		

The study observed a significant association between undernutrition and immunization status (p=0.042). Majority of the undernourished children were not fully immunized (72.6%). Immunization status was found to have a significant association with the number of children in the family (p=0.041). The majority of those children who were fully immunized lived in households with a smaller number of children. Immunization status was significantly associated with the child's age (p=0.001). Lower aged children were fully immunized as compared to older aged children (mean age of fully immunized children was 29±7 months). About (73.5%) of fully immunized children's fathers were educated, and immunization was significantly associated with father's education (p=0.041). (Table 2)

**Table 2:** Association between morbidity conditions and personal hygiene

Variables	Category	Good Personal hygiene		χ^2^	p-value
		Yes	No		
**Skin infection**	Yes	15(31.9)	86(48.6)	4.170	**0.041**
	No	32(68.1)	91(51.4)		
**Fever**	Yes	7(14.9)	62(35)	7.064	**0.008**
	No	40(85.1)	115(65)		
**Cough**	Yes	12(25.5)	88(49.7)	8.790	**0.003**
	**No**	**35(74.5)**	**89(50.3)**		

## Discussion

Undernutrition was high among children of illiterate mothers (63.8%) and the children of working mothers were affected by more morbidities (96.6%) as compared to children of mothers who were homemakers.

Morbidity was also found to be high among children belonging to families with low income (66.1%) and low socio-economic backgrounds (93.1%). Similar findings have been reported by Tada Y et al^19^, Abuya BA et al^20^, and Safikul Islam et al^21^ where the prevalence of underweight children was consistent with this study.

The most common morbidities among the children were skin infection (45.1%), fever (30.8%), cough (44.6%), pneumonia (27.7%) and diarrhoea (24.1%). These findings are similar to the findings of studies in other settings by Adhikari D et al^22^, Srivastava DK et al^23^, Taffa N et al^23^. These morbidities were associated with lack of personal hygiene, mother's age, her education and occupation, family income and type of household which are findings similar to other studies (24–26). In the Indian context, a study conducted by Ukey UU et al^25^ in urban slums of Visakhapatnam showed a higher prevalence of morbidity conditions (82%) among under-five children.

A study conducted in Nepal reported a considerably higher prevalence (60%) of underweight children in urban slums^21^. Also, a study in the urban slums of Etawah^22^ showed a prevalence of undernutrition at 37.1% among children of mothers in the age group of 20–35 years that is consistent with the present study where the mean age of mothers of under-nourished children was found to be 22.9±4.04.

In this study, 38.8% of the under-five children were fully immunized with the majority (65.1%) being male. BCG vaccination was given to 90.2% of the children but vaccination coverage for measles was low (28.1%). These findings are similar to a study conducted by Sharma R et al^27^ where total immunization coverage was 25%, BCG vaccination coverage was 75%, and measles vaccination coverage was 29.9%. This study also shows that the number of children of younger parents who were fully immunized was more than children of relatively older parents (mean age of fully immunized children's mother was 22.5±4.4 and fathers was 30±6.5).

Majority of the fully immunized children's parents had a lower number of children. Lower aged children were fully immunized as compared to higher aged children (mean age of fully immunized children was 29±7 months). Among fully immunized children, 73.5% & 34.9% of their fathers and mothers were educated respectively. This was found to be consistent with a study conducted among 746 rural to urban migrant mothers with children aged up to 2 years by Kusuma YS et al.^29^ It was also found that mother's age; educational status; the frequency of health care use; head of the family's education, job and income were significantly associated with full immunization coverage.

A study conducted in an unregistered slum of Mumbai by Banerjee J et al^30^ showed that 43% of mothers did not have an immunization card recording the immunization status of their child which is consistent with the present study.

This study was not without its limitations. Morbidity status was based on self-reported signs and symptoms which were not confirmed by clinical examinations or diagnostic tests. Immunization status might be under-reported as the immunization card was used to verify immunization status in this study. Many of the parents could not produce the card at the time of data collection. Only weight for age was used to assess the health status of children in this study. Several respondents refused to give written consent although they were ready to participate and gave verbal consent.

Healthcare needs to be made accessible for mobile and migrant slum populations without the hassle of documentation. Provision of basic needs, adequate and safe drinking water supply, sanitary facilities, mosquito nets and shelter to improve life in the slums need to be prioritized as they represent a specific vulnerable group.

## Conclusion

Overall, in our study, family characteristics including parental education, occupation and income were found to be statistically significantly associated with childhood outcomes. Undernutrition was high among children of illiterate mothers and less than half of the under-five children were fully immunized. The availability of safe drinking water and sanitation and the use of mosquito nets to prevent vector-borne diseases were basic needs that need to be urgently met to improve child health.

## Figures and Tables

**Figure 1:**
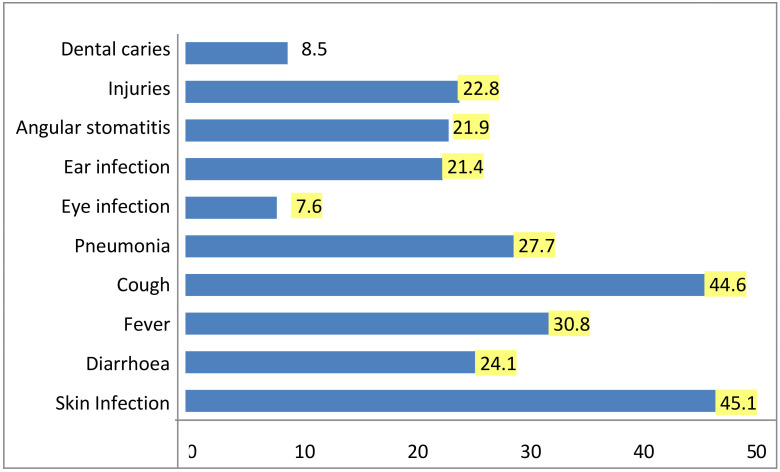
Proportion of under-five children by morbidity based on general and physical examination (Multiple conditions reported) n=174

**Table 3:** Association between socio-demographic factors and childhood undernutrition

Variable	Category	Undernutrition		χ^2^	p-value
		Yes	No		
**Mother's education**	Literate	17(23.3)	55(36.4)	3.893	**0.048**
	Illiterate	56(76.7)	96(63.6)		
**Monthly income (Rs.)**	>5000	57(78.1)	85(56.3)	10.069	**0.002**
	<5000	16(21.9)	66(43.70		
**Type of household**	Semi-pucca	2(2.7)	24(15.9)	8.299	**0.004**
	kutcha	71(97.3)	127(84.1)		
**Adequate water supply**	Yes	54(74.0)	128(84.8)	3.7	**0.051**
	No	19(26.0)	23(15.2)		
**Number of children in** **the family**	Mean±SD	2.5±4.2	2.8±1.1	4.26*	**0.041**
**Number of under five** **children in the family**	Mean±SD	1.43±0.04	1.3±0,4	3.390*	**0.060**
**Child's age**	Mean±SD	13.29±7	35.9±1	188.469*	**0.001**
**Father's education**	Literate	61(73.5)	84(59.6)	4.596	**0.041**
	Illiterate	22(26.5)	57(40.4)		

* Fisher's *exact*

χ^2^ – Chi-square test

**Table 4:** Association between socio-demographic factors and immunization status

Variables	Category	Immunization		χ^2^	p-value
		Yes	No		
**Number of** **children in** **family**	Mean±SD	2.5±4.2	2.8±1.1	4.26	**0.041**
**Child age**	Mean±SD	13.29±7	35.9±1	188.469*	**0.001**
**Father's education**	Literate	61(73.5)	84(59.6)	4.596	**0.041**
	Illiterate	22(26.5)	57(40.4)		
Total		83(100)	141(100)	224	
